# Effects of Ground Conditions on Microbial Cementation in Soils

**DOI:** 10.3390/ma7010143

**Published:** 2013-12-27

**Authors:** Daehyeon Kim, Kyungho Park, Dongwook Kim

**Affiliations:** 1 Department of Civil Engineering, Chosun University, 375, Seosuk-Dong, Dong-Gu, Gwangju 501-759, Korea; E-Mail:dkimgeo@chosun.ac.kr; 2 Department of Civil and Environmental Engineering, Incheon National University, Incheon 406-772, Korea; E-Mail: munhakng@nate.com

**Keywords:** microbe, CaCO_3_, SEM, XRD, EDX, cementation, ground condition

## Abstract

The purpose of this study is to understand the effect of ground conditions on microbial cementation in cohesionless soils. Since the method of microbial cementation is still at the experimental stage, for its practical use in the field, a number of laboratory experiments are required for the quantification of microbial cementation under various ground conditions, such as relative densities, relative compactions and particle size distributions. In this study, in order to evaluate the effectiveness of microbial cementation in treated sands and silts, an experiment was performed for different relative densities of silica sands, for different relative compactions of silts and for different particle size distributions of weathered soils sampled from the field. Scanning electron microscope (SEM), X-ray diffraction (XRD), energy dispersive X-ray (EDX) spectroscopy and mapping analyses were implemented for the quantification of the levels of microbial cementations for sand, silt and weathered soil specimens. Based on the test results, a considerable microbial cementation was estimated depending on the soil conditions; therefore, an implementation of this new type of bio-grouting on a weak foundation may be possible to increase the strength and stiffness of weak ground.

## 1. Introduction

### 1.1. Background and Objective

The grouting technique, a typical treatment method for soft ground, is used for strength improvement and the prevention of underground water flow. Specifically, the grouting technique is implemented to increase strength and to decrease the permeability of soft ground under dams, slope reinforcement, embankments and landfill structures. However, these ground treatment techniques do not offer effective solutions for secondary or tertiary environmental issues caused by the depletion of natural resources, the pollution of underground water and post-construction maintenance. Chemical or cement grouting techniques may be a more effective method of ground improvement, but people should be aware of the seriousness of the environmental issues that may be caused by the use of traditional chemical grouting. The materials used in chemical and cement grouting produce a large amount of carbon dioxide and other air and water pollution during their manufacturing and implementation processes. Following the worldwide trend of a low-carbon green-growth policy, the government and private companies also have set new policies, regulations and strategies to reduce greenhouse gases and other hazardous emissions. Therefore, extensive research should be conducted to find suitable substitutes for these typical, hazardous grouting materials.

The purpose of this study is to understand and to analyze the effects of ground conditions on microbial cementation (calcium carbonate deposition by microbes). Since the method of microbial cementation is still in the experimental stage, a number of laboratory experiments are required under various ground conditions.

### 1.2. Literature Review

There are several previous studies regarding the implementation of microbes in soil cementation, but their research scopes are still limited. The previous studies on microbial cementation are summarized as follows: Experimental studies for the identification of the microbial cementation mechanism in sands were conducted, followed by a visual comparison of microbial cementations in sands under different microbe concentrations [1–4]. It was found that the cemented sand after bacteria treatment with a high concentration of microbes had a much higher strength compared to that of the cemented sand treated with a lower concentration of microbes (Kim *et al.* [1]). For the bacteria cementation, microbes were extracted from centrifugation and were mixed with culture fluid, reactive solution (calcium chloride, CaCl_2_, solution) and sands. Based on the pocket penetrometer test, the strengths of bacteria-treated sands after seven days of curing were measured up to 0.5 MPa.

Kim *et al.* [2] identified the effectiveness of microbial treatment in reducing the permeability of sands and silty sands from the constant head permeability test results. It was found that the permeability (*k*_non-treated sands_ = 2.27 × 10^−2^ cm/s and *k*_non-treated silty sands_ = 4.52 × 10^−4^ cm/s) of the non-treated sands and non-treated silty sands dropped to 6.30 × 10^−3^cm/s (approximately a 30% decrease) and 1.57 × 10^−4^ cm/s (approximately a 35% decrease), respectively.

Similar studies were conducted in the United States, Australia and the Netherlands. Mitchell *et al.* [5] and Dejong *et al.* [6] analyzed the applicability of bio-grouting on weak ground, varying the microbe sizes and soil particle sizes to increase the strength of soft ground. Paassen *et al.* [7] also conducted an experimental study on bio-grout, which implemented micro-organisms by ureolysis in granular soils to increase both the stiffness and strength of the granular soils. Wijngaarden *et al.* [8] performed numerical and mathematical studies to implement microbial-induced carbonate precipitation on ground improvement.

Dejong *et al.* [6] identified the cementation levels among soil particles of a non-treated sand specimen, a lime-treated sand specimen and a microbe-treated sand specimen. However, these tests were conducted for loose (relative density, *D*r, around 35%) and collapsible sand specimens. Relative density, *D*r, is a number between 0% and 100%, indicating how dense sandy soils are. Relative densities, *D*r, of zero and 100% correspond to the possible loosest and densest states of a given sand. The test results revealed that cementations were visible within a few days from the microbe-treated sand specimen; however, its successful implementation in practice still remains questionable, because of the lack of ease of the periodic injection of microbes and the versatile geological conditions in the field.

Ivanov *et al.* [9] described bio-cementation using microbes as a new field in geotechnical engineering. They claimed that bio-cementation reduces the permeability of soils, due to bio-clogging, and improves the mechanical properties of the soils, such as the strength and stiffness of the soils. However, most studies on microbial grouting engineering have been conducted for the understanding of complex knowledge related to microbiology, ecology, Earth chemistry and ground engineering; therefore, more serious research is required for the proposal of reasonable bio-related cementation methods that can be implemented in the actual construction field.

Based on large-scale test results, Paassen *et al.* [7,10] succeeded in enhancing the soil strength near the surface using bio-grouting. They: (1) prepared a larger concrete soil tank (8 m in length × 5.6 m in width × 2.5 m in height); (2) laid a thin sand layer of 25 cm; and (3) installed 48 seismic sensors and geotextiles on the side of the soil tank. The soils used in the experiment were poorly-graded sands sampled from the Itterbeck quarry. For the tests, three inlets and three outlets were installed to simulate a hydraulic gradient of 0.3 in the vertical direction. As a result, the strength of the soil was significantly improved after the bio-grouting technique was implemented.

Qabany *et al.* [11] used ureolytic bacteria to implement microbial-induced precipitation on the soil improvement method and measured S-wave velocity to estimate the calcite precipitation effect in the laboratory. It was found that the calcite precipitation amount was increasing approximately linearly with an increasing S-wave velocity.

## 2. Microbial Cementation

### 2.1. Soil Void Ratio and Microbe Size

Soil selection is an important factor influencing microbial growth. Soils are mainly classified into four categories: gravel, sand, silt and clay. Generally, it was reported that the most active microbial reaction occurs in sands [1–11]. In this study, the microbial solution (a mixture of microbes and culture fluid) and reactive solution (CaCl_2_ solution) was mixed with sands, silts and weathered soils separately to investigate the effect of soil conditions on the microbial reaction.

The relationship between microbe size and void ratio is also an important factor for microbial growth. Therefore, in this study, three different ground conditions in terms of relative density (*D*r = 40%, 60% and 80%) for the sands, relative compaction (RC = 60%, 75% and 90%) for silts and particle size distribution (well-graded and poorly-graded) for weathered soils were prepared to evaluate the effects of void ratio and soil particle sizes on microbial calcium carbonate deposition.

The typical microbial size is of the order of 1–3 μm. For the successful implementation of microbial deposition, the microbes in the mixture of the reactive solution and microbial solution should be optimally sized to be able locate between the soil voids without difficulties. Void sizes larger than 3 μm or smaller than 1 μm may result in a reduced effect of calcium carbonate deposition. In this study, the microbe, *B. pasteurii* (named Korean collection for the type culture (KCTC) 3558 by the Korean collection for the type culture of the Korean Research Institute of Bioscience And Biotechnology) is used, because it can pass through the voids of soil particles when the reactive solution and the microbial solution are injected into soils. *B. pasteurii* is an aerobic microbe that has been proven to produce calcium carbonate well within cohesionless soils in the previous studies [1–4]. In addition, it is reported that the sizes of the created calcite cemented particles could be approximately 20 times the size of the microbe when extracellular calcite precipitation occurs [6]. Thus, the cemented particles created cannot be held between the particles for soils having very large voids, as the cemented particles would fall through the soil particles.

### 2.2. Reaction between Microbe and Urease

For the preparation of the bio-grouting solution, in this study, the *B. pasteurii* microbes of KCTC 3558 were cultivated for 24 h in a shaking incubator of 180 rpm at 30 °C at the biological resource center of Chosun University. Under the microbe cultivation environment, the concentration level of microbe was approximately 10^7^ colony forming units per milliliter (CFU/mL). Then, centrifugation (6000 rpm at 4 °C for 6 min) was applied on 1000 mL of the microbe fluid to obtain 25 mL of high-concentration microbe fluid, whose microbe concentration level is approximately 4.0 × 10^8^ CFU/mL.

Separately, 1 L of culture fluid was made by mixing eight grams of nutrient broth (which consists of 5 g enzymatic digest of gelatin and 3 g of beef extract) with 20 g urea (H_2_NCONH_2_) and by adding purified water until the total solution volume became 1 L. The enzymatic digest of gelatin and beef extract are the microbial growth elements in which substances required for growing plants are dissolved. Urea is a colorless crystalline compound, which is highly reactive with water. Urea is the end product of protein metabolism of all mammals and some fish, and ammonia is created by the protein breakdown. Urease is an enzyme catalyzing the reaction for hydrolyzing urea to create ammonia and carbon dioxide. In other words, Urea is hydrolyzed into a carbonate (CO_3_^2−^) and two ammonium ions (NH_4_^+^), as follows:

$$\text{CO}{\left({\text{NH}}_{2}\right)}_{2}+2{\text{H}}_{2}\text{O }\overset{\text{Urease reaction}}{\to }{\text{CO}}_{3}^{2}{}^{-}+2{\text{NH}}_{4}^{+}$$ (1)

The carbonate (CO_3_^2−^) is combined with the calcium ion (Ca^2+^) dissolved in the aquatic solution of calcium chloride (CaCl_2_) to result in calcium carbonate (CaCO_3_) depositions:

$${\text{Ca}}^{2+}+{\text{HCO}}_{3}^{{}^{-}}+OH^{-}\to {\text{CaCO}}_{3}↓+{\text{H}}_{2}\text{O}$$ (2)

## 3. Laboratory Experiment

Soil cementation by microbes contributes to the deposition of calcium carbonate (CaCO_3_) in the voids between soil particles through a microbial urea reaction. The calcium carbonate is tightly filled between voids and acts as a bond between the particles; therefore, the strength of soils is increased. In this study, as mentioned in the previous section, the relative densities (40%, 60% and 80%) of sands, relative compactions (60%, 75% and 90%) of silts and the particle size distribution (well-graded and poorly-graded) of weathered soils were changed to identify the effects of soil particle sizes and voids on microbial cementation (or the creation of calcium carbonate).

For the identification of the effectiveness of microbial cementation, scanning electron microscope (SEM), X-ray diffraction (XRD) and energy dispersive X-ray (EDX) devices were used. In addition, mapping analysis was also implemented for a visual comparison of the amounts of the calcium carbonate created. The amount of calcium carbonate created under each test condition was measured after 7 days of curing (or mixing soils with the microbial solution and the reactive solution).

### 3.1. Soils Used in This Study

**Figure 1.**
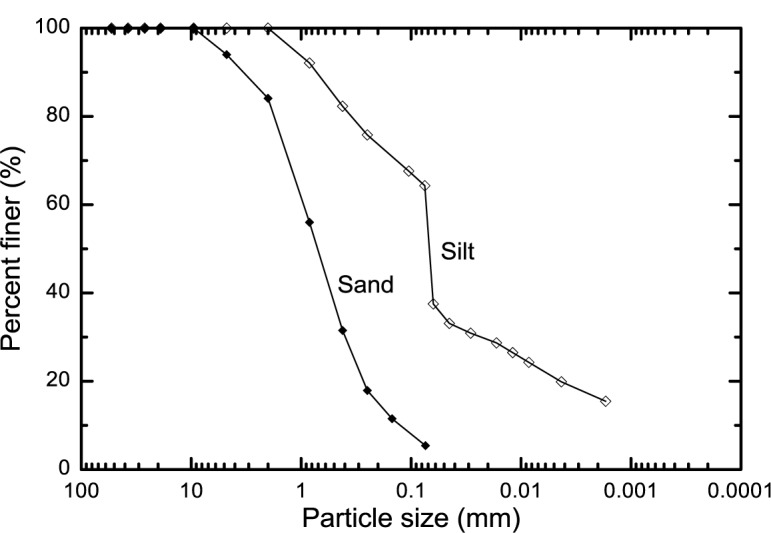
Particle size distributions of sand and silt used in the study.

In this study, Jumunjin sand was used. The sand was classified as “poorly-graded sands and gravelly sands” based on the Unified Soil Classification System (USCS), and its classification symbol is denoted as “SP”. Artificially made silts were used in the experiment. The silt was grouped as “inorganic silts, very fine sands, rock flour, silty or clayey fine sands (symbol = ML)”, according to USCS. The silt specimens were prepared using the soil passing No. 10 sieve (the grain size passing the 2 mm sieve). The physical properties of the sands and silts are summarized in Table 1, and their particle size distributions are provided in Figure 1.

The silt was compacted following the A-compaction method proposed by the ASTM standard (ASTM D 2487-11 [12]). The diameter and volume of the compaction mold are 10 cm and 1000 cm^3^, respectively. During the laboratory test, the mold was attached to a base plate at the bottom and to an extension at the top. The soils were mixed to produce different water contents and then were compacted in three equal-thickness layers using a hammer that delivers 25 blows to each layer. The mass and drop height of the hammer were 2.5 kg and 30 cm, respectively.

**Figure 2.**
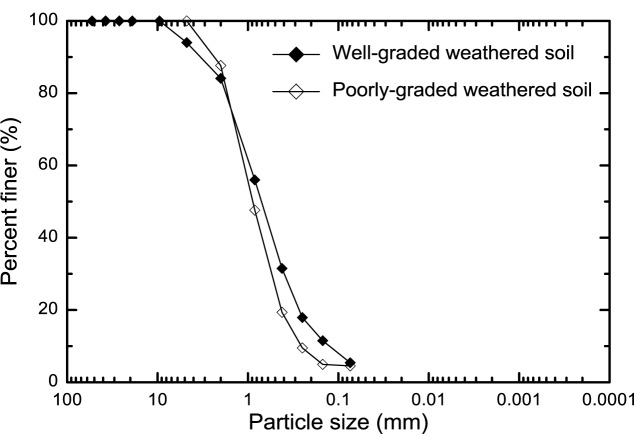
Soil particle size distributions of poorly-graded and well-graded weathered soils.

The two different weathered soils were obtained from the field. From the sieve analyses of these two different weathered soils, one soil was classified as symbol “SW” (well-graded sands and gravelly sands, little more of coarse or no fines) and the other soil grouped as “SP” (poorly-graded sands and gravelly sands, little or no fines) based on the USCS soil classification system. The soil particle distributions of the poorly-graded and well-graded weathered soils are shown in Figure 2. The uniformity coefficients (*C*_u_) and coefficients (*C*_g_) of curvature were 3.9 and 0.77 for the poorly-graded weathered soil and 7.7 and 1.22 for the well-graded weathered soil, respectively.

### 3.2. Sample Preparation

#### 3.2.1. Sand Specimen

In order to conduct the laboratory test to identify the amount calcium carbonate creation depending on the relative density, the target relative densities were set to 40%, 60% and 80%. The soil specimens were made following the mixing ratios of sand, calcium chloride solution and microbial solution shown in Table 2. To examine the effect of sand relative density on calcium carbonate deposition, a consistent amount ratio (1:1) of the microbial solution to the calcium chloride solution was assumed. The sand specimens with different relative densities were prepared as follows: For the pre-obtained maximum and minimum void ratios (*e*_max_ = 0.754 and *e*_min_ = 0.517), the known volume of the specimen mold and the target relative densities, the corresponding required dry soil weights (γ_d_) for the target relative densities of 40%, 60% and 80% were calculated. Then, the corresponding total specimen unit weights (γ_t_) were obtained from the following equation:

$$\gamma_{\text{t}}=\;\gamma_{\text{d}}\left(1+\frac{\text{OMC}(\%)}{100}\right)$$ (3)

where OMC is the optimum moisture content in %.

The difference between γ_d_ and γ_t_ is the amount of water required to obtain the maximum compaction; however, in this study, the amount (48 g ≈ 4 mL) of the sum of solutions (calcium chloride solution and microbial solution) is assumed to be equal to the water amount required to produce the maximum compaction. For each sand specimen, the sand weight given in Table 2, 24 mL of calcium chloride solution and 24 mL of microbial solution were evenly and well mixed. The mixture was placed in the mold carefully in multiple layers manually by tapping the sides of the mold to obtain the uniformity of the target relative density.

#### 3.2.2. Silt Specimen

**Figure 3.**
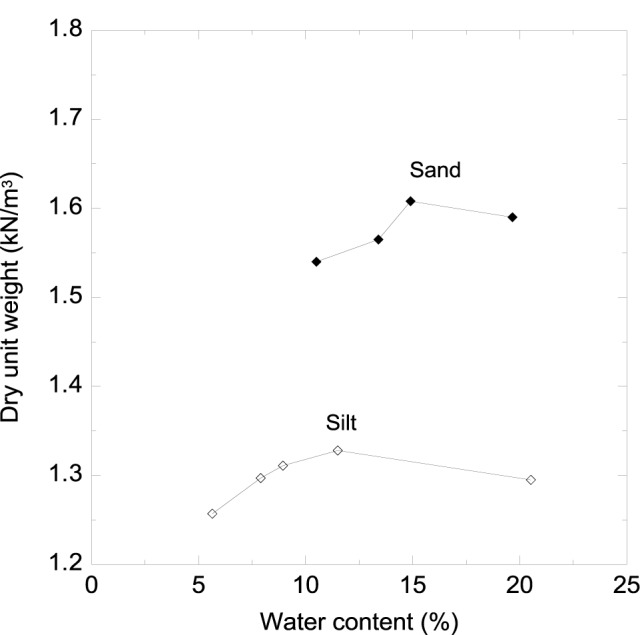
Compaction curves of sand and silt used in the study.

Silt specimens were prepared with the mixing ratios of silts, calcium chloride solution and microbial solution proposed in Table 2. The target relative compactions were set to 60%, 75% and 90% to identify the amount of calcium carbonate depositions depending on the levels of compaction. The relative compaction is defined as the percentage ratio of the soil sample density (or field density) to the maximum density obtained from the standard compaction. The preparation method of silt specimens is different from that of sand specimens. From the maximum dry density (γ_dmax_) in the compaction curve shown in Figure 3, the dry unit silt weights (γ_d_) corresponding to the target relative compactions of 60%, 75% and 90% were calculated (Table 2). Then, 11 mL of calcium chloride solution and 11 mL of microbial solution are added to the silts, and these were evenly mixed to have uniformity in terms of relative compaction level.

#### 3.2.3. Weathered Soil Specimen

Since the previous tests were done only for the poorly-graded Jumunjin sand (which is considered as a standard sand in Korea) and the artificially made silts, the applicability of microbial cementation in weathered soils should also be examined. In Korea, weathered soils are dominant throughout the country. Two different weathered soils were collected. The maximum and minimum void ratios of soils were 0.899 and 0.588 for weathered soil classified as “SP” and 0.777 and 0.521 for the other weathered soil classified as “SW” respectively. Using their maximum and minimum void ratios, the amounts of dried soil weights (γ_d_) were calculated (Table 2). The optimum moister contents (OMCs) for these two soils are different. Therefore, for a consistent comparison of the effects of the particle size distribution on the microbial cementation, the same amounts of calcium chloride solution (24 mL) and microbial solution (24 mL) were added and mixed for both dried weathered soils.

### 3.3. Brief Description of Experimental Equipment

The SEM scans across the specimen surfaces uses an electron lens and provides enlarged images for which any viewer can identify the images with ease. The EDX analyzer is attached to the SEM equipment, and they are used in combination. The EDX enables nondestructive analytical identifications of the structure and chemical composition of minerals contained in a specimen. For these identifications, the XRD equipment examines the directions and scattered intensity of a diffracted X-ray by calculating the kinetic energy from the collision of high-speed X-ray. The EDX analyzer conducts an element analysis of a sample surface based on the characterization of the different weight structures of the elements. In addition, mapping analysis provides an image of distribution (or scatters) of particular elements existing on the sample surface.

## 4. Result and Discussion of Experiment

### 4.1. SEM and XRD Analyses

**Figure 4.**
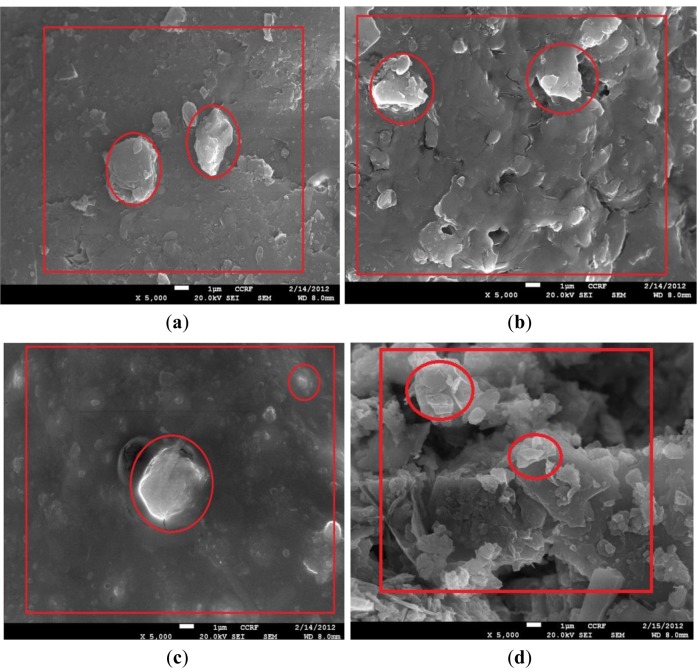

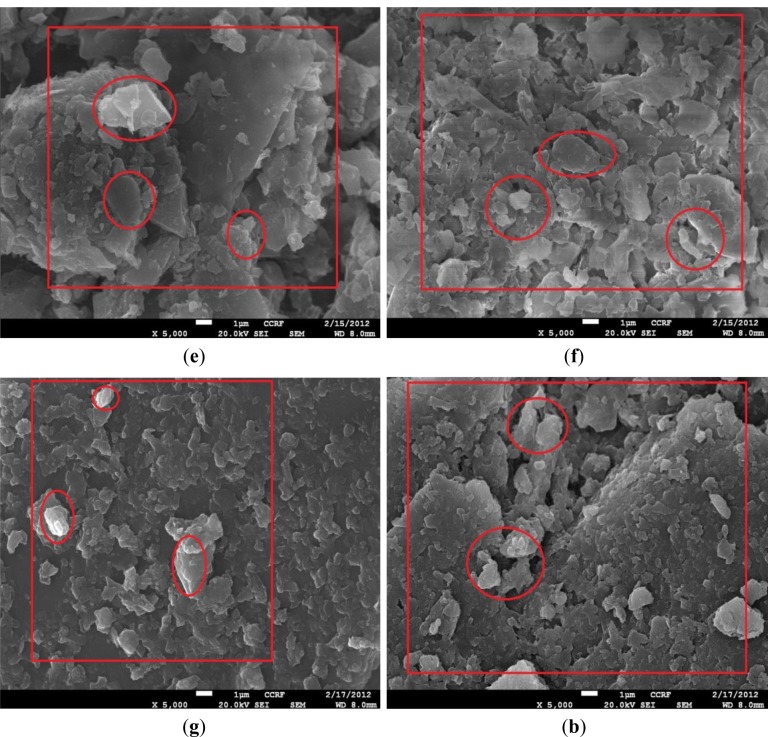
Results of SEM (the magnified image is 5,000 times larger than its original scale) of sand specimens with (**a**) *D*r = 40%; (**b**) *D*r = 60% and (**c**) *D*r = 80%; silt specimens with (**d**) RC = 60%; (**e**) RC = 75% and (**f**) RC = 90%; and (**g**) well-graded, and (**h**) poorly-graded weathered soil specimens.

The SEM produces two-dimensional data, similar to three-dimensional images, that allows visual identification of depositions created on the soil particle surfaces. For the analyses using SEM, EDX, XRD and mapping, all the microbe-treated samples were dried at 121 °C to have zero water content. The results of SEM analysis provide 5000 times magnified views of each tested specimen, as shown in Figure 4. It was identified that the calcium carbonate depositions (typically, the color of calcium carbonate in SEM is close to white) by microbes were observed (for example, the red circles of Figure 4). Calcium carbonate deposition was also confirmed by the results of XRD analysis. From the results of XRD analyses of all the sand, silt and weathered soil specimens, compounds of quartz (SiO_2_), microcline (KAlSi_3_O_8_) and calcite (CaCO_3_) were identified, and an additional compound of albite (NaAlSi_3_O_8_) was also identified in both sands and weathered soils.

### 4.2. EDX and Mapping Analysis

**Figure 5.**
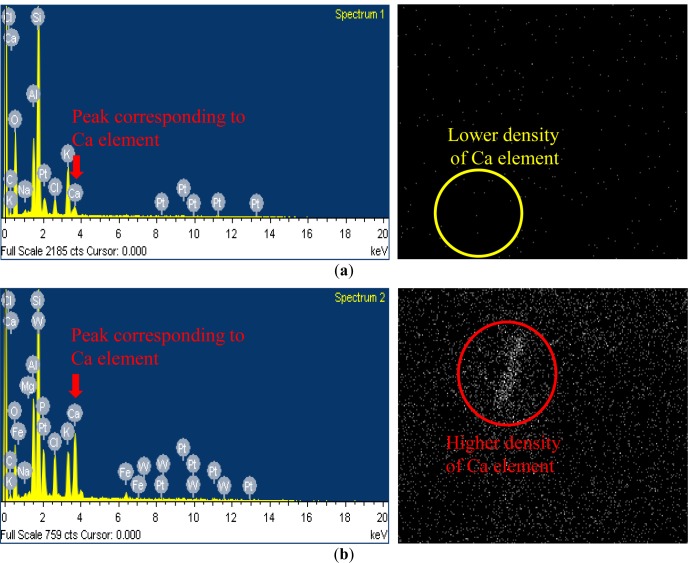
Energy dispersive X-ray (EDX) and mapping analysis (the magnified image is 5000 times larger than its original scale) results of specimens with (**a**) minimum Ca element observation (well-graded weathered soil specimen) and (**b**) maximum Ca element observation (poorly-graded weathered soil specimen).

The EDX belongs to the electronic microscope family. It is analysis equipment used to analyze which elements are included in a small sample. The EDX analyzer records the counts of representative elements from the elements’ spectrum. The EDX analysis result provides a graph having the count numbers of the representative elements on the y-axis and the energy levels of the corresponding counts on x-axis, as shown in the left figures of Figure 5. In Figure 5, the EDX and mapping analyses results are provided only for the specimens with minimum and maximum Ca element observations. For each plot, the maximum scale of the y-axis is set to be the maximum number of counts from the spectrum of the representative elements. Then, specifically coded software can be used to convert the numbers of counts of the elements into the weight percentage (wt%) of the elements.

**Figure 6.**
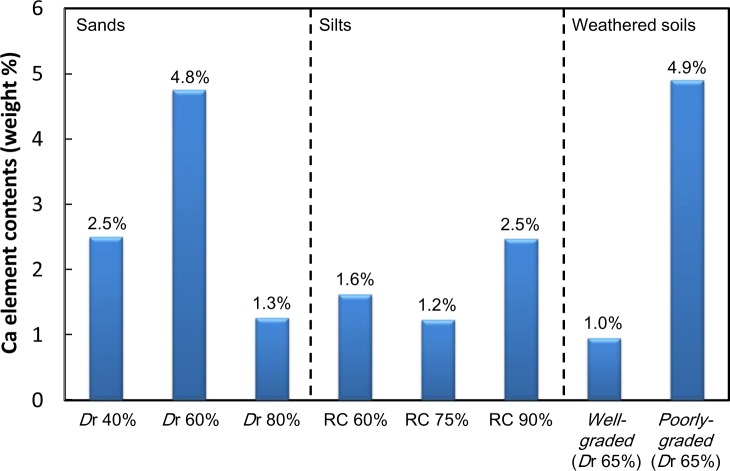
Ca contents (by wt%) depending on the ground condition.

Direct measure of the CaCO_3_ deposition amount is difficult. In our previous studies [1–4], for the validation of CaCO_3_ deposition, we mixed the microbial solution and the reactive solution without soils and identified that the minerals created were pure calcium carbonate from the XRD analysis. Although the use of XRD results for the identification of the calcium carbonate creation amount is not the best assessment, we intended to indirectly identify the level of calcium carbonate depositions by examining the Ca element content by weight using the given equipment available in this study. Figure 6 shows the indirect (or relative) comparison of the amounts of Ca content (Ca element wt%) among other elements contained in all the specimens. In this study, it is assumed that the amount of CaCO_3_ deposition is approximately proportion to the Ca element content. Mapping is an analysis device that shows the distribution of the element of interest from the SEM photos magnified 5000 times. The right figures of Figure 5 show the detected Ca components on the soil surfaces in each area, which is the same in the corresponding area shown in the SEM result in Figure 4. Large numbers and higher clarities of white points of a certain area indicate that Ca components are more concentrated in the area. As mentioned in the previous sentences, the indirect quantifications of Ca components from the figures are shown as EDX plots. Therefore, the mapping analysis also contributes to the improved reliability of the SEM and EDX results.

In comparison to Ca content among the sands with relative densities of 40%, 60% and 80%, the sand specimen with the target relative density of 60% exhibited the highest calcium carbonate deposition, as shown in Figure 6. It is inferred that soil particles are not reasonably combined under either somewhat loose (*D*r = 40%) or dense (*D*r = 80%) conditions; therefore, less calcium carbonate depositions were created.

The highest calcium carbonate deposition rate among the silt specimens (under different relative compactions of 60%, 75% and 90%) was observed from the silt specimen with the target relative compaction of 90%. It was found that higher compaction levels contributed to higher calcium carbonate depositions. However, their differences were not significant. Further studies should be done to clarify the difference of the amount of Ca depending on different compaction levels of silts.

With respect to the particle size distribution effects on the calcium carbonate deposition, the EDX analysis revealed that more calcium carbonate depositions were found from the weathered soil specimen with a poorly-graded distribution. The result implies that the higher calcium carbonate depositions were observed for more uniform voids, as expected for the poorly-graded soils.

In summary, from the examination of calcium carbonate deposition analysis results for all the samples tested in this experiment, it was overall found that the microbial cementation is more active in sand specimens than in silt specimens. In addition, more microbial cementation is found for the weathered soils that were poorly-graded than for those with well-distributed weathered soils. The calcium carbonate deposition amount from the poorly-distributed weathered soil specimen is about five times more than that from the well-distributed weathered soil specimen. Therefore, it was identified that the effects of void ratio and void size on microbial cementation are significant. For the given test results, more microbial cementation was observed for a higher void ratio and void size. However, in this study, it was not possible to examine the microbial cementation of looser samples and larger void sizes. Further research is required to find more effective combinations of void ratio and void size for different types of soils that produce more microbial cementation.

## 5. Conclusions

This study aimed to identify the creation of calcium carbonate and the characteristics of cementation of soil depending on the ground conditions for sand, silt and weathered soil specimens. The basic physical properties were identified for different relative densities (40%, 60% and 80%) of sands, relative compactions (60%, 75% and 90%) of silts and particle size distributions (well-graded and poorly-graded) of weathered soils. Calcium carbonate depositions were identified based on the results of the SEM, XRD, EDX and mapping analyses. Based on their results, the following conclusions were drawn.

1. The mineral created from microbial cementation was identified as calcium carbonate (CaCO_3_) based on XRD analysis results, and the CaCO_3_ was observed on the surface of soil particles, as can be seen in the SEM photographs. In addition, Ca content (by wt%) and distribution of the Ca element were examined using EDX and mapping analyses to improve the reliability of the test results.
2. The amounts of CaCO_3_ created for the sand specimens were about two times more than those for silt specimens. It is inferred that the somewhat wider voids among the soil particles of sands contribute to a greater amount of microbial cementation; however, additional research should be conducted to find the optimal void size and ratio that maximize the microbial cementation.
3. From the test results of weathered soils with different soil particle size distributions, it was identified that more active calcium carbonate deposition by microbes was observed in poorly-graded distributions. This indicates that more uniform voids among soil particles result in more active calcium carbonate deposition from the laboratory test results.

The method used for the estimation of the amount of calcium carbonate created in this paper was selected based on the given environment (devices and equipment) of the university. However, for more accurate estimation of the amount of calcium carbonate creation, the use of a combination of an electron probe X-ray micro analyzer (EPMA) and of thermo gravimetric analysis (TGA) is recommended. The authors will conduct further analysis based on improved measuring equipment in further research.

## Figures and Tables

**Table 1.** Physical properties of the soils used in this study. USCS, Unified Soil Classification System.

Soil Type (USCS Symbol)	Specific Gravity (G_s_)	% Passing No. 200 Sieve	Maximum Dry Unit Weight (γ_dmax_)	Optimum Moisture Content (O.M.C)	Liquidity Limit (LL)	Plasticity Index (PI)
Sand (SP)	2.67	2.4%	1.577 kN/m^3^	14.9%	N.P.*	N.P.*
Silt (ML)	2.67	64.3%	1.303 kN/m^3^	11.6%	23.3%	N.P.*

* N.P. indicates “non-plastic.”

**Table 2.** Specimen mixing ratios of sand, silt and weathered soil specimens. *D*r, relative density; RC, relative compaction.

Specimen (USGS symbol)	*D*r or RC	Soil (g)	Calcium chloride solution (mL)	Microbial solution (mL)
Sand (SP)	*D*r 40%	298.8	24	24
	*D*r 60%	307.6	24	24
	*D*r 80%	316.9	24	24

Silt (ML)	RC 60%	223.0	11	11
	RC 75%	279.3	11	11
	RC 90%	297.4	11	11

Weathered Soil (SP)	*D*r 65%	261.4	24	24

Weathered Soil (SW)	*D*r 65%	279.3	24	24
